# Effects of Green Apple (Golden Delicious) and Its Three Major Flavonols Consumption on Obesity, Lipids, and Oxidative Stress in Obese Rats

**DOI:** 10.3390/molecules27041243

**Published:** 2022-02-12

**Authors:** Ilknur Selek Aksoy, Semih Otles

**Affiliations:** Food Engineering Department, Ege University, Bornova, İzmir 35040, Turkey; semih.otles@gmail.com

**Keywords:** Golden Delicious, flavonol, lipid, obesity, oxidative stress, quercetin

## Abstract

Obesity is becoming increasingly common all over the world and global strategies are accordingly being developed to prevent it. In order to support the strategies, the effects of green apple (Golden Delicious) and the consumption of its three major flavonols (quercetin-3-glucoside, quercetin-3-D-galactoside, and quercetin-3-rhamnoside) on body weight; the weight of liver, kidney, and spleen; some lipid parameters in serum; and total lipid ratios of liver and kidney and oxidative stress parameters of obese rats were studied. This study was conducted on two experimental groups: one of which was given an apple, and the other was given flavonols, in addition to their high-energy diet; along with a sham and a control rat group, for 4 weeks. According to results, there was no difference in body and organ weights between groups. The liver and kidney weights increased in obese rats, but there was no difference between the total lipid ratios in these organs. The addition of green apple and selected flavonols to the high-energy diet of rats was not sufficient to prevent the increase in body and organ weights, but it supported the reduction in some lipid fractions and in oxidative stress parameters of obese rats. Moreover, this study supported the argument that obesity causes most of the lipid fractions increase in serum and induces oxidative stress.

## 1. Introduction

Overweight and obesity are defined as abnormal or excessive fat accumulation that presents a risk to health. Overweight and obesity are major risk factors for a number of chronic diseases, including diabetes, cardiovascular diseases, and cancer. Once considered a problem only in high income countries, overweight and obesity are now dramatically on the rise in low- and middle-income countries, particularly in urban settings. This situation not only effects mature women and men, but also children and the young population. Obesity causes a decrease in life quality and increased mortality rates, and is also a factor effecting national economies in a negative way [1,2].

There is a need for personal lifestyle changes and/or pharmacological therapies for the treatment of obesity. Pharmacological therapies and surgical interventions are generally not appropriate; although, they may be administered in some cases. The use of natural herbal supplements in the treatment of many diseases, including obesity, is a growing area. Today, natural and alternative anti-obesity agents are used in the treatment of obesity. This may reduce the side effects of chemical anti-obesity agents. Unfortunately, in addition to the short-term benefit of drug treatment in obesity, there is usually a weight gain after discontinuing the medication, and it also has side effects. It has been reported that pharmacological options have potential damage and long-term use causes cardiovascular problems. In general, other methods used by the public in weight loss are plants, vitamins, herbal supplements, and changing eating habits [3,4,5,6].

Foods that provide weight control have begun to be investigated in the mid-19th century when industrialization led to the spread of obesity. For years, people have been trying to lose weight by avoiding some food groups such as carbohydrates and with certain additives such as cabbage soup, vinegar, or grape juice. Recently, scientists have stated that foods such as green tea, dairy products, and cinnamon support their weight control by affecting the fat and/or carbohydrate metabolism at the intracellular level. Tools such as caffeine and green tea, which can be used in obesity management, have become the recommended strategies for weight loss and control. These components may increase energy consumption and reduce the metabolic rate during weight loss [5,7].

The obesity epidemic has costly consequences. One of them is the quest for reliable weight regulator application. There are individual approaches such as diet clubs, commercial diet programs, online diet sites, and sports programs, but not enough to stop the weight gain epidemic. Complementary and Alternative Medicine National Institutes of Health Institutes support the research of nutrients, fibers, and phytochemicals in a variety of health-friendly plants. Consumers’ interest has shifted to non-medical treatment methods due to the suspicion of food support contaminants, agricultural trade practices, and the side effects of weight loss pills [7].

Global strategies focus on nutritional guidelines and lifestyle changes, such as limiting calorie intake and increasing physical activity, to slow down the development of obesity. A food science research has revived interest in the potential of natural products to counteract obesity. These products include high potential phytochemical feeding for health promotion and disease prevention. Notably, the use of polyphenols has been expanded in animal models and in vitro studies because of their comparatively negligible adverse effects. Flavonoids, which are the most abundant subclass of polyphenols found in the human diet, have suggested potential protective effects against obesity and other metabolic diseases in numerous reports. Among them, quercetin (QT) is one of the most abundant polyphenolic flavonoids, which is present in fruits and vegetables and displays many biological, health-promoting effects in a wide range of diseases [8,9,10,11].

The combination of multiple phytochemicals can result in a synergistic effect that increases their bioavailability and movement to multiple molecular targets, thus providing advantages over chemically-containing treatments. The anti-obesity effects of these components are mediated by the regulation of various metabolic pathways including fat absorption, the increase in lipolysis and the decrease in lipogenesis, and the differentiation and proliferation of adipose tissue precursors [9].

In many studies, obesity and oxidative stress effects have been observed with the addition of some plants and/or antioxidants to the diets of obese rats fed with a high fat diet. Although the feeding period varied between 4 and 16 weeks, the results of the parameters were observed in each study [12,13,14,15,16,17]. In this study, various parameters of obese rats, which were added green apple and apple primary flavonols to their diets for 4 weeks, were evaluated based on the interest in natural products against obesity.

The apple was preferred because the apples were widely consumed for general purpose and weight loss in Turkey, are rich in phytochemicals, and epidemiological studies correlated apple consumption with the reduction in some cancers, cardiovascular diseases, asthma, and diabetes risk. In laboratory studies, it was found that apples have a very strong antioxidant effect that prevents the proliferation of cancer cells, decreases lipid oxidation, and lowers cholesterol. Apples contain various phytochemicals including quercetin, catechin, fluoridzine, and chlorogenic acid, all of which are potent antioxidants. Apples are one of the main sources of flavanoid nutrition that show the strongest relationship with decreasing mortality rate. It is second in the total concentration of phenolic compounds, and perhaps more importantly, apples have the highest free phenolic fraction compared to other fruits. This means that these components are not bound to other components in fruits, and therefore the absorption of phenolics in the blood is better [18].

While there are more than 6000 apple varieties in the world, it is emphasized that very few types of apples are of commercial importance. The commercially produced varieties include Golden Delicious, Red Delicious, Fuji, the Gala groups, and Granny Smith. The most produced apple varieties in Turkey are Starking, Golden, Starkrimson, and Amasya apples [19].

In this study, we aimed to investigate the green apple effect, Golden Delicious type was preferred. The effects of green apple (Golden Delicious) and primary flavonols (quercetin-3-glucoside, quercetin-3-D-galactoside, and quercetin-3-rhamnoside) of green apple consumption on the body weight of obese rats; the weight of certain organs (liver, kidney, and spleen); lipid ratios in serum, liver, and kidney; and oxidative stress parameters were studied. In many prior studies on obese patients, signs of oxidative stress in their bodies increased and protective antioxidant enzymes decreased. For this reason, obesity is considered to be a condition that leads to inflammation and chronic oxidative stress. Therefore, the activity of antioxidant enzymes on the control and trial groups will be determined. Changes in major antioxidant enzymes will be observed, including in SOD, GPx, and CAT, and also in MDA concentrations, a product and marker of lipid peroxidation, which is the common approach in measuring oxidative stress [18,20].

Atomic Absorption Spectroscopy (AAS) will be utilized to determine the trace elemental composition of apple based on the thought that a possible reduction in oxidative stress may be caused by the content of the antioxidant as well as the various antioxidant enzymes and their co-factor minerals. With this aim, the amount of the SOD co-factor in foods will be determined, including manganese and copper, iron as a catalase co-factor, and selenium for glutathione peroxidase. In conclusion, it will be researched whether green apple and its primary flavonols are a solution for some obesity parameters.

## 2. Results

### 2.1. Mineral Element Analysis

The amount of manganese and copper were determined as the SOD co-factor, iron as a catalase co-factor, and selenium for glutathione peroxidase because of the thought that potential difference in oxidative stress parameters may be caused by the content of antioxidant enzymes’ cofactors as well as the antioxidant. The amount of minerals mentioned above in green apple is shown in Table 1. The mineral substance analysis results of the apple were found to be close to the literature [21,22,23]

### 2.2. High-Performance Liquid Chromatography Analysis

**Figure 1:**
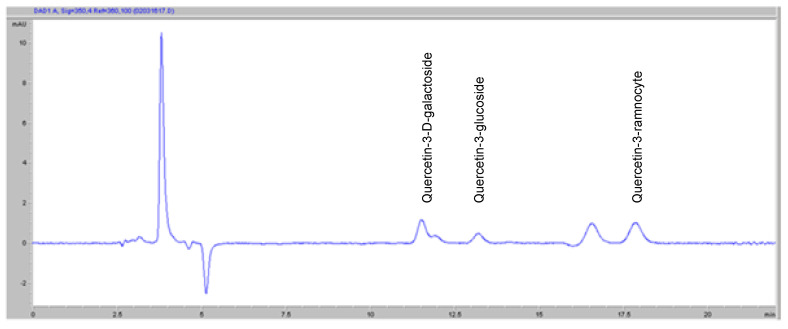
Quantities of selected minerals and flavonols in green apple.

In the case of Golden Delicious, quercetin-3-D-galactoside, quercetin-3-rhamnoside, and quercetin-3-glucoside were among the most common quercetin compounds. Random samples were selected from apples. They were extracted by an ultrasonic method and their amounts were determined by HPLC. Quantities of selected antioxidants in samples are shown in Table 1 and chromatogram are shown in Figure 1. The quantities of quercetins of the apple were found to be close to the literature [24,25,26].

### 2.3. Determination of Body and Organ Weights

**Figure 2:**
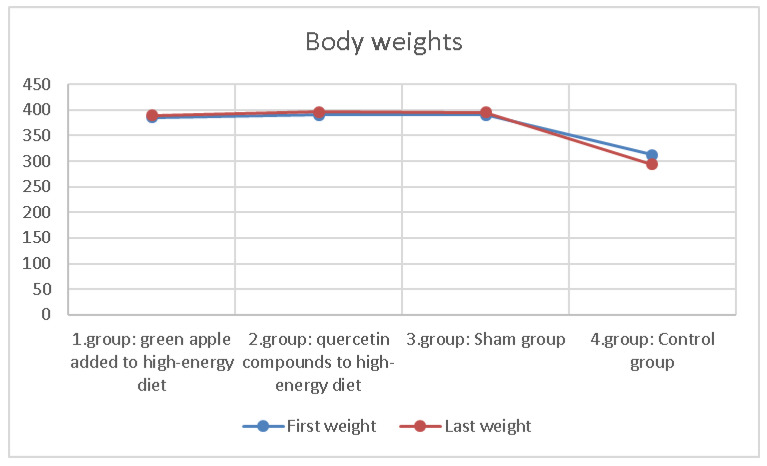
Comparison of first and last weights averages of 10 rats in groups.

The body weights of the rats in the groups were measured every week. The differences between the first week and last week weights are shown in Table 2 and Figure 2. In addition, desire for food and water in groups was also observed. It was observed that the desire for feed consumption was higher in the group that added apples to their diet.

There was no difference in body weight differences between groups statistically. The rats in the group with apples added to their diet were observed to tend to feed more. 

**Figure 3:**
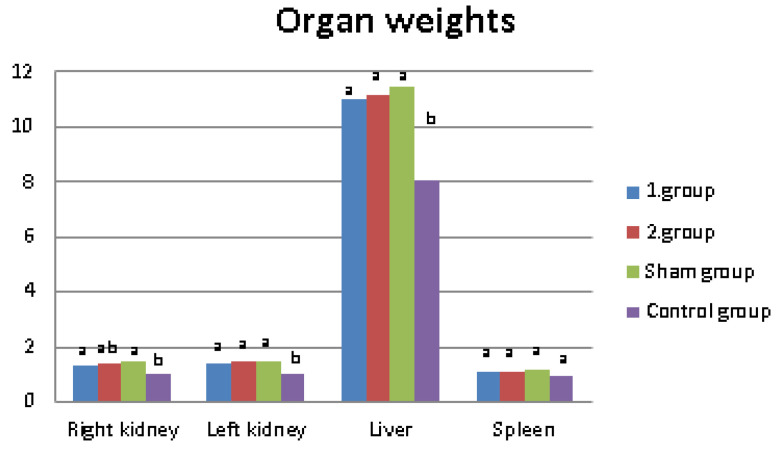
Comparison of organ weights averages of 10 rats in groups. ^a,b^ Means in the same group with unlike superscripts differ significantly (*p* < 0.05).

One of the aims of this study was to determine the effect of green apple and quercetins on liver, kidneys, and spleen weights. In addition, total lipid amounts of liver and kidneys measured to determine whether obesity, green apple, or quercetin has effects on organ lipid ratios. The results are shown in Table 3 and Figure 3. According to our results, the addition of apple and quercetin to the high fat diet did not affect the organ weights. Although spleen weights were not affected by obesity, liver and kidney weights were higher in obese. In addition, the results showed that the liver and kidney weights increased in obese rats, and there was no difference between the total lipid ratios in these organs.

### 2.4. Determination of Lipid Ratios

Free fatty acid, phospholipid, serum triglyceride, and total cholesterol concentration were determined in each serum sample from rats. The results are shown in Table 4. 

The rate of free fatty acids, phospholipids, and triglycerides in the sham and control groups were statistically different. The control group had lower concentrations of free fatty acid, phospholipid, and triglyceride concentrations in serum. Moreover, quercetins and green apple were effective in decreasing these three lipid concentrations. The concentrations of free fatty acid and phospholipid in the serum of the group that added apple to their diet were lower than that of quercetins. Total cholesterol concentrations of the sham and control groups were the same. Moreover, the addition of apple and quercetins to the diet did not change the total cholesterol level.

### 2.5. Determination of Oxidative Stress Parameters

To understand quercetins effect on antioxidant enzymes, SOD activity, CAT activity, GPx activity, and MDA concentration were determined. The results are shown in Table 5.

The SOD, GPx activities, and MDA concentrations in the sham and control groups were statistically different. In terms of these three parameters, the sham group has lower antioxidant enzyme activity. According to the results of our study, obesity is not effective on the catalase activity. Moreover, the addition of apple and quercetins to the diet does not change the catalase activity in the erythrocytes. The addition of apple and quercetins to the diet contributed to the increase in GPx activity and decrease in MDA concentrations.

## 3. Discussion

Obesity, following a rising trend all over the world, is associated with many chronic health problems as well as decreasing quality of life, leading to deaths, and it is also a factor that has a negative effect on national economies. Global strategies are developed to slow down the development of obesity via lifestyle changes. Nevertheless, obesity patients may choose a way to lose weight using a variety of chemical or herbal products without changing their daily lifestyles and eating habits [1,2,9]. Food studies that can support preferences of obese patients have been carried out for years. Recently, scientists have stated that foods such as green tea, dairy products, and cinnamon support weight control by affecting the metabolism at the intracellular level [5,7]. Based on these studies, we aimed to observe the effects of green apple (Golden Delicious) and primary flavonols (quercetin-3-glucoside, quercetin-3-D-galactoside, and quercetin-3-rhamnoside) of green apple consumption on the body weights of obese rats. Apples are widely consumed for weight loss in Turkey, are rich in phytochemicals, and epidemiological studies have shown that apple consumption correlates with the reduction in some cancers, cardiovascular diseases, asthma, and diabetes risk. In addition, the other aim was to determine the weight of certain organs (liver, kidney, and spleen), lipid ratios (in serum, liver, and kidney), and oxidative stress parameters because studies in humans and most animal models have shown that the development of obesity causes lipidosis in classical adipose tissues, other tissues, and organs, and oxidative stress [27,28].

According to our results, there was no difference in body weight changes between groups statistically. The rats in the group with apples added to their diet were observed to tend to feed more. The reason could be the high rate of fructose in the apple. It was found that apple has a high fructose content, with 104.01 mg/g on a fresh weight basis [29]. However, the content of sorbitol (17.94 mg/g) and sucrose (15.82 mg/g) in apple fruits were much lower; similar results were found by [30,31]. In same study, they found that the amount of fructose in peach was 5.79 mg/g, in watermelon was 36.94 mg/g, and in cherry was 84.65 mg/g. The main administration of fructose was shown to decrease hypothalamic satiety signaling and increase feeding in animals. Moreover, fructose compared to glucose results in greater food-cue reactivity within brain reward regions and increases the motivation for food rewards. Compared to glucose, fructose ingestion results in smaller increases in circulating levels of insulin, leptin, and glucagon-like polypeptide-1, hormones that increase satiety [32]. Moreover, fructose has a slower absorptive rate and produces less satiety than other sugars [33]. 

In terms of organ weights, we found that spleen weights were not affected by obesity; however, liver and kidney weights were higher in obese rats and this result supports the results of many studies. A reduction in hepatomegaly in overweight patients who lose weight was observed [34], and it was concluded that total liver weight was increased (36%, *p* < 0.05) in high-fat diet rats in comparison with the control group [35]. In a study aiming to investigate the effect of early obesity on kidneys, kidney weight in obese dogs was found to be 31% more than normal weight ones [36]. In addition, it was mentioned that obesity in mice is accompanied by changes in organ weight and certain changes are the enlargement of the liver, kidneys, and heart. The addition of apple and quercetin to the high fat diet did not affect the organ weights. This may have been due to the amount of apple and quercetin added to their diet. New studies may be conducted by adding a higher rate to the diet [37].

Although the liver and kidney weights increased in obese rats, there was no difference between the total lipid ratios in these organs. Some studies have been summarized in the literature on the non-lipid-related increase in liver and kidney weights, and in consideration of these, the results of our study may be due to these reasons: In one study, it was remarked that hepatomegaly occurring in an obese or in a diabetic patient may be the result from the presence of glycogen accumulation in the liver [38]. In another study, it was concluded that the toxic potential of imidacloprid results in increased liver weight of high-dose-exposed animals, and, ultimately, they showed the liver of mice exposed to the high imidacloprid treatment evidenced congestion and fatty degeneration. They also found higher doses of imidacloprid increased the weight of kidney with tubular changes showing its nephrotoxicity in mice [39]. Moreover, it was concluded that obesity and weight gain increase the risk of kidney stone formation [40]. 

Lipid results in this study showed that obesity contributes to the increase, and quercetins and green apple to the decrease, of free fatty acid, phospholipid, and triglyceride concentrations in serum. The concentrations of free fatty acid and phospholipid in the serum of the group with added apple to their diet was lower than that of quercetins. This result can be interpreted as other compounds in the apple other than quercetins, which reduces the free fatty acid and phospholipid concentrations. These components are thought to be pectin and dietary fiber found in apples because they concluded that pectin in the apple reduces lipid peroxidation and the apple fiber reduces the phospholipid ratio in serum [41,42]. According to the results of our study, obesity and the addition of apple and quercetins to the diet are not effective on the total cholesterol level. In the literature, there are studies conducted on the effect of apple, polyphenols in the apple, and flavonols on lipids in serum. They can show different results from each other and from our study. Among the potential factors involved in such heterogeneity are: (i) factors inherent to the individuals: (epi) genetic factors, gut microbiota, baseline conditions (body mass index, medication), sex, health status, ethnicity, and age; and (ii) factors intrinsic to the type of study (design, duration, dose, and type of product) [43]. In one study, it was investigated whether apple polyphenols (dimers to pentadecamers of procyanidins, phenolic acids, phloretin glycosides, monomeric flavan-3-ols, and others) had an effect on lipid-related plasma profiles. They organized three different groups: (1) diet containing 5% apple polyphenol, (2) diet containing 0.5% apple polyphenol, and (3) the control group. They concluded that lipid-related plasma profiles showed no statistical differences in terms of triglyceride, total cholesterol, and NEFA [44]. Moreover, in another study, the efficacy of 12-week intake of polyphenols extracted from apples (600 mg/day) on moderately obese male and female subjects was evaluated. They confirmed that 12-week ingestion of polyphenol-containing capsules significantly decrease total cholesterol levels [45]. In another study, the influence of apple pomace (AP) and apple juice concentrate (AC) supplementation on body weight and fat loss as well as lipid metabolism in obese rats fed a high-fat diet were investigated. Diet-induced obese rats were assigned to three groups (n = 8 for each group): high-fat diet (HFD) control, HFD containing 10% (*w*/*w*) AP, and HFD containing 10% (*w*/*w*) AC. There was also a normal diet group (n = 8). After 5 weeks, body weight gain, white adipose tissue (WAT) weight, serum total cholesterol, low-density lipoprotein cholesterol and triglyceride concentrations, epididymal adipocyte size, and lesion scores were significantly lower, and serum high-density lipoprotein cholesterol concentration and brown adipose tissue weights were significantly higher in the AP and AC groups compared with the HFD group [46]. In terms of free fatty acid, it was concluded that the basal release of FFA from adipose tissue to meet lean body mass energy needs is greater in upper-body obese women than lower-body obese or nonobese women. Elevated NEFA concentrations in obesity are thought to arise from an increased adipose tissue mass [47,48,49]. González-Sarrías and workmates confirmed that the flavonol-containing tea products are effective regulators of blood cholesterol (total, LDL, and HDL) as well as of body mass index and waist circumference. The cocoa or apple products were effective at reducing total- and LDL-cholesterol, and the cocoa products were also able to significantly decrease the levels of triacylglycerides. These results might suggest that the metabolic regulatory efficacy of these three flavonol-containing products could be ranked as tea > cocoa > apple but caution should be taken with this interpretation due to the differences in the number of studies carried out with each source of flavonols as well as the differences in the doses and the composition of the products. Further studies are needed to corroborate this comparison [43]. 

In our study, there was no difference in glutathione peroxidase activities, SOD activities, and catalase activities among the experimental groups adding green apple and antioxidants to their diet. Therefore, it could be concluded that mineral substances, which are determined as cofactors of antioxidant enzymes, have no effect in these amounts. In terms of SOD, GPx activities, and MDA concentrations, the control group has higher antioxidant enzyme activity. This result shows that obesity is a condition that leads to chronic oxidative stress in accordance with most of the studies. However, CAT values obtained in our study differ from some studies. It was found that the activation of SOD, GPx, and CAT in the overweight children increased, but their activities are depleted in obese children in an article on this topic. The activation of these antioxidant enzymes in overweight children may be to counteract the effect of the oxidative stress generated by reactive oxygen species (ROS). The excess production of ROS with insufficient antioxidant enzymes in obese children may have a serious adverse effect on red blood cell membranes, resulting in lipid peroxidation enhancing production of MDA concentrations [20]. In another article, it was observed that erythrocyte glutathione (GSH) levels and catalase activity were reduced and MDA levels were enhanced in obese rats when compared to controls [50]. Amirkhizi and colleagues hypothesized that obesity is an independent risk factor for lipid peroxidation and decreased activity of cytoprotective enzymes in humans. To test this hypothesis, they measured the concentration of plasma MDA, erythrocyte CuZn-SOD, GPX, and CAT activities. They found that there was a significantly positive correlation between body mass index (BMI) and plasma MDA. On the other hand, women with healthy BMI had significantly higher erythrocyte CuZn-SOD and GPx activity than obese women. No significant difference was observed between two groups in erythrocyte CAT activity as result of our study [51].

Although the addition of apple and quercetins to the diet did not support an increase in SOD activity, it contributed to an increase in GPx activity and decrease in MDA concentrations, but they were not sufficient to bring them to the same levels as non-obese rats. In another study, it was shown that a significant increase in TRAP values and a significant decrease in the MDA values in rats fed with apple peel and pulp added to basal diet and non-oxidized cholesterol was found [52]. In addition, it was demonstrated that treatments with either quercetin or quercetin–iron complexes alleviated oxidative stress in obese rats, by reducing oxidant markers and increasing antioxidant defense. This elevation in antioxidants may be due to the neutralization of ROS as quercetin has been found to scavenge superoxide anions and other free radicals in vitro [50].

This study indicated that apple and quercetin glycosides were not effective on weight loss. It was observed that there is a greater desire to consume feed in the apple-consuming group and it is surmised that this was due to the fructose content in the apple. When the organ weights were evaluated, it was seen that the addition of apple or quercetin to the diet did not make any difference. In addition, it was concluded that obesity had no effect on spleen weights but increased liver and kidney weights. There was no difference between the obese group and the control group in terms of liver and kidney fat ratios, indicating that the weight increases were non-lipid related.

The results of this study show that obesity causes most of the lipid fraction increases in serum and induces oxidative stress. The addition of apple and quercetin glycosides to the high-energy diet was not sufficient to prevent the increase in body and organ weights, but it was shown to support the reduction in some lipid fractions and oxidative stress parameters.

## 4. Materials and Methods

### 4.1. Materials

Golden Delicious was used because it is the most produced green apple variety in our country, Turkey [19]. As it can be found in the market every season and due to some physical changes during storage, it was obtained from a constant supplier in Izmir during the analysis. The apples were used in the analysis as a whole with the skin and flesh. The average skin amounts of the apples were determined as 10%, and this weight was taken into consideration in all analyses. 

Quercetin 3-D-galactoside (Sigma-Aldrich 00180585, St. Louis, MO, USA), quercetin-3-rhamnoside (Sigma-Aldrich 00740580), and quercetin-3-glucoside (Sigma-Aldrich 00140585), were purchased from St. Louis, Mo, USA, are the most common quercetin compounds in the Golden Delicious species, the amounts of 3 quercetin compounds in green apple were determined and used in rats’ feed. 

### 4.2. Animals

All animal experiments were carried out in accordance with Turkey Regulation on Clinical Trials, 2014 and Turkish Republic Health Ministry Good Clinical Practices Guide, 2015. Experimental procedures were approved by the Animal Ethics Committee of Süleyman Demirel University (AEC NO: 21438139-604.01.02-31).

The 16-week-old male Wistar Albino type, 30 obese and 10 normal weight rats were obtained from the Süleyman Demirel University Experimental Animal Production and Research Laboratory. The rats were divided as normal and obese by calculating the Lee Index values. Lee index is expressed as cubic root of body weight in grams divided by the naso–anal length in mm multiplied by 104. If Lee index value is greater than 300, the rat is obese [53]. Feeding and decapitation operations were carried out in the same laboratory. The reason we prefer male rats was to eliminate the antioxidant effect of estrogen in female rats.

We divided 20 of the obese rats at random into 2 groups of 10 rats each, which were named the experimental groups and fed on the high-energy diet and test components. A further 10 obese rats were separated into a sham group and fed only with a high-energy diet of the same content as the experimental groups. Finally, 10 normal weight rats were separated as control group and fed with standard rat feed during the experiment.

The feed given to the control group had 19.2% crude protein, 5.12% crude cellulose, 4.48% fat, and 4.6% ash; the feed given to the obese groups had 28.5% crude protein, 4.21% cellulose, 12.5% fat, and 8.06% ash. High-energy diet contained 8% more calories, 48% more protein, and 179% more fat than normal diet.

Rats were exposed to standard light (12 h daylight/12 h dark) and heat (25 °C). Each group was fed with enough ad libitum water and feed. Green apple was added to the diets of one of the experimental groups in 3% of their feed by gavage once a day. Antioxidant blend containing 0.008% quercetin-3-glucoside, 0.02% quercetin-3-D-galactoside, and 0.016% quercetin-3-rhamnoside, which corresponded, respectively, to their concentrations in a 3% green apple diet, was given to the other experimental group by gavage once a day. Apple and antioxidants were dissolved in 10% ethanol, 10% tween 80, and 80% saline solution [54]. In addition, solute given to control and sham groups by gavage once a day.

At the end of the 4 weeks, the experiment was terminated by decapitation under anesthesia with 10% ketamine (Alfamin, Alfasan IBV.) and 2% xylazine (Alfazin, Alfasan IBV.). After the rats were decapitated, the kidneys, liver, and spleen will be weighed individually in each rat and the effect of the food and antioxidants on the organ weights were observed. Liver and kidney of each rat were stored at −80 °C in phosphate buffer (1/10 *w*/*v*) to determine lipid ratios. Serum and hemolysates prepared were stored at −80 °C until the analysis.

### 4.3. Mineral Element Analysis

The assays were performed on Analytikjena contrAA 700 brand Atomic Absorption Spectroscopy, Analytikjena GMBH, Jena, Germany, C_2_H_2_-air flame. The wavelength used to determine the iron content is 248 nm, 324 nm for copper, and 279 nm for manganese.

For the extraction of minerals (Fe, Cu, and Mn) from the samples, a previously used method [55] was modified. Firstly, 5 g of green apple was weighed, dried at 80 °C, pre-burned into the crucible, and burned until completely white. The resulting ashes were dissolved in 10 mL of 1% HCl and filtered to give atomic absorption spectroscopy.

In addition to the 3 minerals mentioned above, selenium contents of samples were determined. As the method of analysis, the NMKL-161 method, which is within the scope of TURKAK Accreditation, was used. In summary, after the pre-treatment in the microwave oven, the amount of selenium was determined by atomic absorption spectrophotometer.

### 4.4. High-Performance Liquid Chromatography Analysis

Antioxidants from apple samples were extracted by the method used in [56]. Samples were extracted by ultrasonic method at room temperature and in the absence of light with methanol. The sample was extracted with 10 mL of methanol for 1 h, 10 mL for 30 min, and then 5 mL for 30 min. The three extracts were combined to a final volume of 25 mL. Solutions to be analyzed by HPLC-DAD (Agilent, Santa Clara, CA, USA) were filtered through a membrane filter (5 µm pore size) prior to injection.

Chromatographic separation by HPLC was carried out with isocratic elution [57]; 10 µL volume sample was given to the system at a flow rate of 0.8 mL/min at 350 nm. Aqueous 40% methanol solution containing 1% acetic acid was used as the mobile phase.

In the case of Golden Delicious, quercetin 3-D-galactoside, quercetin 3-ramnocyte, and quercetin 3-glucoside are the most abundant quercetin compounds. Therefore, the amounts of these 3 quercetin compounds were determined [57,58].

### 4.5. Determination of Body and Organ Weights 

Before the rats were decapitated, body weights were measured once a week for 4 weeks of feeding. After the rats were decapitated, the kidneys, livers, and spleens were individually weighed in each rat and the effect of food and antioxidants on the organ weights were observed. 

### 4.6. Determination of Lipid Ratios

Serums from each rat were centrifuged at 825 g for 20 min and 4 °C to determine lipid ratios. Stored at −80 °C until the analysis. Total cholesterol (Sigma-Aldrich Cholesterol Quantitation Kit MAK043), triglyceride (Sigma Serum Triglyceride Determination Kit TR0100), phospholipid (Sigma-Aldrich Phospholipid Assay Kit MAK122), and free fatty acid (Sigma-Aldrich Free Fatty Acid Quantitation Kit MAK044) analysis were performed in each serum sample. 

Liver and kidneys were stored at −80 °C in phosphate buffer (1/10 *w*/*v*) to determine total lipid amounts. The liver and kidneys in phosphate buffer (20 mL) were put into an extraction thimble that contained chloroform/methanol (40 mL; 3:2 *v*/*v*), separately. The obtained mixture was homogenized at 155 rpm for 15 min. Then, water (8 mL) was added, and the mixture was shaken vigorously to facilitate the transfer of oil into the chloroform and other products into the water–methanol layer. The chloroform layer was then separated via a separation funnel and collected. The extraction steps were repeated two times. The obtained chloroform layers were combined and evaporated using an evaporator (65 °C, for 20–30 min.). Then, the trace amount of chloroform was evaporated in drying oven and weighed remaining oil [59].

### 4.7. Determination of Oxidative Stress Parameters 

To determine the parameters of oxidative stress, hemolysates were prepared from whole blood taken from each rat into EDTA tubes. After the whole blood was centrifuged at 4000 rpm for 5 min, the plasma was separated, the remaining erythrocyte package was placed in a 0.5 mL clean glass tube and 2 mL isotonic NaCl was added and mixed. The supernatant fraction was discarded by centrifugation at 4000 rpm for 5 min. The last two steps were repeated 3 times. After the last repetition, 2 mL cold distilled water was put into the tube and erythrocytes exploded. The obtained hemolysates were stored at −80 °C until analysis.

In the erythrocytes obtained; superoxide dismutase (SOD) activity (Cayman Superoxide Dismutase Assay Kit 706002), catalase activity (Cayman Catalase Assay Kit 707002), glutathione peroxidase activity (Cayman Glutathione Peroxidase Assay Kit 703102), and malondialdehyde concentration (Cayman TBARS Assay Kit 10009055) determination were performed.

### 4.8. Statistical Analysis

All results were expressed as mean ± standard error of the mean (SEM, n = 10). One-way ANOVA and Duncan test were used for statistical evaluation and the differences were considered significant at *p* < 0.05.

## 5. Conclusions

This study indicated that apple and quercetin glycosides were not effective on weight loss. It was observed that there was a greater desire to consume feed in the apple-consuming group and it is estimated that this was due to the fructose content in the apple. When the organ weights were evaluated, it was seen that the addition of apple and quercetin to the diet did not make any difference. In addition, it was concluded that obesity had no effect on spleen weights but increased liver and kidney weights. There was no difference between the obese group and the control group in terms of liver and kidney fat ratios, indicating that the weight increases were non-lipid related. The results of this study show that obesity causes most of lipid fraction increases in serum and induces oxidative stress. The addition of apple and quercetin glycosides to the high-fat diet was not sufficient to prevent the increase in body and organ weights, but it was shown to support the reduction in some lipid fractions and oxidative stress parameters. 

## Figures and Tables

**Table 1:** Quantities of selected antioxidants and minerals.

**Antioxidants**	**Miligram/Gram (Dry Matter)**
Quercetin-3-glucoside	0.0194 ± 0.0011
Quercetin-3-D-galactoside	0.0557 ± 0.0007
Quercetin-3-rhamnoside	0.0395 ± 0.0002
**Minerals**	**Microgram/Gram (Dry Matter)**
Iron	15.32 ± 0.27
Copper	1.51 ± 0.03
Manganase	1.88 ± 0.01
Selenium	0

**Table 2:** Body weights (gram) of rats ^†^.

Rat Groups	First Weight	Last Weight	Difference	Observations
1.group: green apple added to high-energy diet	385.6 ± 53.1	388.6 ± 59.5	2.8 ± 15.3 ^a^	Feed consumption is more than the other groups.
2.group: quercetin compounds to high-energy diet	390.2 ± 63.2	395.9 ± 65.7	5.7 ± 9.8 ^a^	No significant differences were observed.
3.group: Sham group	390.2 ± 64.9	394.7 ± 73.7	4.5 ± 17.5 ^a^	No significant differences were observed.
4.group: Control group	312.1 ± 29.4	294 ± 31.5	−18.1 ± 43.1 ^a^	No significant differences were observed.

^†^ The means and standard deviations are for 10 mice. Significant difference at *p* < 0.05. ^a,^ means in the same column do not differ significantly (*p* < 0.05).

**Table 3:** Organ weights (grams) and total lipid amounts of organs in rat groups ^‡^.

Rat Groups	Right Kidney	Left Kidney	Liver	Spleen	Total Lipid Amount of Liver (%)	Total Lipid Amount of Kidneys (%)
1.group	1.29 ± 0.28 ^a,b^	1.36 ± 0.33 ^a^	10.99 ± 1.81 ^a^	1.10 ± 0.46 ^a^	4.65 ± 0.73 ^a^	2.39 ± 0.31 ^a^
2.group	1.38 ± 0.30 ^a,b^	1.44 ± 0.30 ^a^	11.12 ± 1.68 ^a^	1.11 ± 0.34 ^a^	4.34 ± 0.27 ^a^	2.67 ± 0.28 ^a^
3.group	1.48 ± 0.42 ^a^	1.49 ± 0.24 ^a^	11.45 ± 2.39 ^a^	1.20 ± 0.31 ^a^	3.92 ± 0.39 ^a^	3.22 ± 0.17 ^a^
4.group	1.03 ± 0.13 ^b^	0.99 ± 0.16 ^b^	8.08 ± 0.83 ^b^	0.98 ± 0.35 ^a^	2.64 ± 0.36 ^a^	2.70 ± 0.25 ^a^

^‡^ Statistical analyses were applied on each analysis and province, separately. ^a,b^ Means in the same column with unlike superscripts differ significantly (*p* < 0.05).

**Table 4:** Some lipid ratios of rat groups ^§^.

Rat Groups	Free Fatty Acid Concentration (Nanomol/Microliter)	Phospholipid Concentration (Micromolar)	Serum Triglyceride Concentration (Triolein Equivalent)	Total Cholesterol Concentration (Microgram/Mililiter)
1.group	41.39 ± 11.9 ^b^	504.24 ± 57.3 ^c^	0.14 ± 0.03 ^a^	0.2965 ± 0.0017 ^a^
2.group	59.28 ± 6.72 ^a,b^	785.6 ± 41.4 ^b^	0.16 ± 0.07 ^a^	0.2949 ± 0.0015 ^a^
3.group	84.48 ± 15.35 ^a^	1157.28 ± 26.9 ^a^	0.31 ± 0.1 ^b^	0.2962 ± 0.0001 ^a^
4.group	39.6 ± 10.87 ^b^	883.76 ± 21.6 ^b^	0.18 ± 0.07 ^a^	0.2954 ± 0.0008 ^a^

^§^ Statistical analyses were applied on each analysis and province, separately. ^a,b,c^ Means in the same column with unlike superscripts differ significantly (*p* < 0.05).

**Table 5:** Oxidative stress parameters in rat groups ^||^.

Rat Groups	SOD Activity (Unit/Mililiter)	CAT Activity (Nanomol/Minute/Mililiter)	GPx Activity (Nanomol/Minute/Mililiter)	MDA Concentration (Micromolar)
1.group	14.67 ± 4.87 ^b^	315.44 ± 16.09 ^a^	120.21 ± 15.85 ^b,c^	21.16 ± 1.42 ^a,b^
2.group	16.91 ± 1.92 ^b^	314.44 ± 33.55 ^a^	183.38 ± 29.92 ^b^	15.71 ± 2.53 ^b,c^
3.group	14.28 ± 1.18 ^b^	270.23 ± 29.59 ^a^	66.22 ± 5.50 ^c^	23.54 ± 1.32 ^a^
4.group	30.56 ± 2.62 ^a^	317.87 ± 16.97 ^a^	727.40 ± 15.66 ^a^	12.91 ± 1.36 ^c^

^||^ Statistical analyses were applied on each analysis and province, separately. ^a,b,c^ Means in the same column with unlike superscripts differ significantly (*p* < 0.05).

## Data Availability

Not applicable.

## Sample Availability

Samples of the compounds are not available from the authors.
